# A Comparison of the Microstructure, Mechanical Properties, and Corrosion Resistance of the K213 Superalloy after Conventional Casting and Selective Laser Melting

**DOI:** 10.3390/ma16041331

**Published:** 2023-02-04

**Authors:** Jiang Wang, Zhen Wang, Qingxuan Sui, Shurong Xu, Quan Yuan, Dong Zhang, Jun Liu

**Affiliations:** 1 School of Materials Science and Engineering, Central South University, Changsha 410083, China; 2 Jinchuan Group Co., Ltd., Jinchang 737100, China

**Keywords:** K213 alloy, alloy casting, selective laser melting, mechanical properties, corrosion resistance

## Abstract

K213 superalloy was fabricated by conventional casting and selective laser melting (SLM). The microstructures of the two samples were examined, and the mechanical properties and corrosion resistance of these two kinds of K213 alloy were comparatively studied. The results show that segregation of Ti occurs at the grain boundaries of the as-cast alloy, resulting in the formation of MC carbide. Many microcracks were formed in the SLM sample. Premature fracture of the as-cast alloy is caused by the precipitation of the harmful phase (Ti, Mo, Nb)C (MC). The MC carbides and microcracks in the as-cast and SLM alloys, respectively, induce tensile fracture. In comparison, the strength of the SLM sample is greater, while the elongation of the as-cast sample is greater. The oxidation resistance of the SLM sample is better at a high temperature of 800 °C. This is due to the relatively uniform composition and microstructure of the SLM alloy. However, the corrosion rate of the SLM alloy is accelerated during the electrochemical immersion corrosion process due to the existence of microcracks.

## 1. Introduction

The alloy K213 is a precipitation-hardened Fe–Ni–Cr-based superalloy. It is widely used in the manufacture of supercharged turbine and gas turbine blades because of its excellent high-temperature mechanical properties, corrosion resistance, and oxidation resistance [1,2,3,4]. However, the service performance of superalloy parts obtained by traditional casting technology is affected by the low degree of alloy purification due to the difficulty of controlling impurity gases [5,6]. In addition, solidification of conventional casting is slow, and segregation of components can occur, eventually leading to poor mechanical properties [7,8,9,10].

K213 alloy components generally have a complex structure. The pursuit of better mechanical properties of components, higher production efficiency, and low cost propels the exploration of net forming technology. Selective laser melting (SLM), as a new method in 3D printing technology, has the characteristics of high manufacturing accuracy, uniform microstructure, and outstanding performance [11,12,13,14,15]. Li et al. [16] studied the peak temperature, molten pool width, and depth in the SLM process of IN625 nickel-based superalloy by using the finite-element method and established a multifactor coupling model. Their experimental results are compared with those previously published. The relative error in simulated peak temperature and melt pool geometry from the surface heat source model is much greater than in the volumetric heat transfer model. In addition, a hybrid heat source model combining the surface thermal model and the volume thermal model was established to realize the high-precision numerical simulation of IN625 SLM. Chang et al. [17] studied the effect of heat treatment on the microstructure and mechanical properties of the SLM-formed GH4099 alloy, revealing the relationship between microstructure and yield strength. The densification behavior and sintering mechanism of the K213 alloy were investigated by metal injection molding and hot isostatic pressing [18]. The precipitated phases and mechanical properties of the alloy were analyzed, and the results showed that the mechanical properties of the metal injection molding alloy were superior to those of the as-cast K213 alloy. The reason is that the grain size is refined, and the γ′ phase and MC carbide are precipitated after aging treatment, which effectively improves the mechanical properties of the alloy. Until now, there are few studies comparing the microstructure analysis and mechanical properties of the K213 alloy as-cast- and SLM-formed.

In practical service environments, especially in high-temperature, complex stress, and seawater conditions, K213 superalloy parts are susceptible to corrosion. In general, corrosion weakens the mechanical properties of the alloy, leading to premature failure of the workpiece [19,20]. Hu et al. [21] studied the high-temperature oxidation and electrochemical corrosion properties of the SLM-formed IN738 alloy. It was found that the SLM-formed alloy has a double-layer protective oxide film structure (the outer layer is composed of Cr_2_O_3_ and a small amount of TiO_2_, and the inner layer is a dense and complete Al_2_O_3_ layer), which has better corrosion resistance than the traditional forming alloy. Luo et al. [22] found that the grain boundary of the Inconel 718 alloy fabricated by a laser additive was preferentially oxidized during high-temperature oxidation. The consumption of Cr and the enrichment of Ti in the interdendritic region can lead to the formation of Cr_2_O_3_, which is beneficial to reducing the corrosion rate. In addition, the primary precipitates in the nickel alloy can also form a countercurrent diffusion channel to improve the oxidation kinetics. However, the high-temperature oxidation and electrochemical corrosion properties of the as-cast and SLM-formed K213 alloy have rarely been studied.

In this work, the microstructure of the K213 alloy produced by conventional casting and by SLM was studied, and the mechanical properties, high-temperature oxidation, and electrochemical corrosion properties of the two samples were analyzed. The purpose is to compare these two different forming techniques and explain the relationships among the manufacturing process, microstructure, and properties of the K213 alloy.

## 2. Materials and Methods

### 2.1. Material Preparation

**Figure 1:**
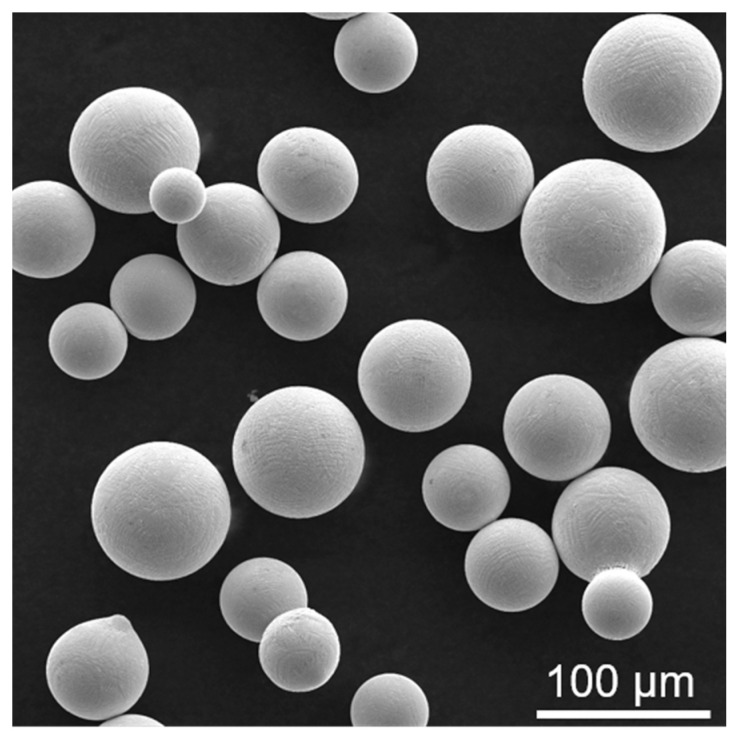
SEM image of K213 alloy powder.

The K213 alloy powder used in the SLM experiment was obtained by a plasma rotating electrode process. The composition is within the nominal specification of the K213 alloy. Figure 1 shows an SEM image of the powder. The K213 alloy powder has regular spherical morphology. It can be seen that the powder has no defects such as hollows and pores. The powder size is in the range of 30–80 μm. Before the SLM, the K213 alloy powder was dried in vacuum for 4 h in order to remove the moisture adsorbed on its surface. An SLM-forming tester (model S210) was used with a continuous 67° scanning strategy. In the SLM process, the substrate was preheated to 100 °C, the laser beam spot diameter was 100 μm, the power was 270 W, the scanning speed was 1150 mm/s, the scanning spacing was 90 μm, and the powder layer thickness was 40 μm. The chemical composition of the K213 alloy obtained by SLM is shown in Table 1.

For a comparative study, conventional casting experiments of the K213 alloy were carried out. First, a master alloy was added into a precision casting vacuum furnace. It was melted under the condition of high-power electrification for 20 min, reaching a fully melted state. The melting temperature reached 1500 °C, followed by a temperature drop to 1450 °C and rapid pouring into the cavity, which was preheated to 980 °C. Finally, the cast K213 alloy was heat-treated at 1100 °C for 4 h.

### 2.2. Tensile and Corrosion Tests

Specimens for tensile and corrosion testing were cut from an as-cast K213 ingot and an SLM alloy by wire cut electrical discharge machining. The tensile properties of the two samples were tested at room temperature by an AGS-X electronic universal testing machine, with a tensile speed of 0.2 mm/min. Each sample was tested three times to confirm the repeatability of test results. The tensile direction of the SLM specimen was perpendicular to the deposition direction during SLM formation.

In order to study the high-temperature oxidation properties of as-cast and SLM samples, 5 × 5 × 2 mm cuboids were processed by wire cut electrical discharge machining. The samples were ground, polished, cleaned with alcohol, and then dried. Each sample was put into a corundum crucible for an oxidation test at 800 °C for different times. The relationship between mass gain and time of the two samples was tested by a static oxidation discontinuous weight gain method.

The electrochemical properties of the two samples were tested by a CHI 660e electrochemical workstation. A three-electrode system was used, and the exposed area was immersed in a 3.5 wt.% NaCl solution after polishing and cleaning. During the corrosion process, the polarization curves were measured at a scanning rate of 0.02 V/s in the scanning range of −2~0 (V).

### 2.3. Characterization

In order to observe the microstructure of SLM and conventional casting specimens, both specimens were first mechanically ground and then polished. They were then wiped for 5 s in a corrosive solution of 2 mL concentrated nitric acid + 2 mL concentrated hydrochloric acid + 1 mL distilled water. In order to accurately observe the composition characteristics of grains and grain boundaries, the samples were ultrasonically cleaned in acetone and alcohol solution in turn. A scanning electron microscope (SEM) was used to observe the microstructure of conventional casting and SLM processing samples, and energy dispersive spectroscopy (EDS) was used for mapping analysis. The tensile fracture and high-temperature oxidation morphology were also observed by an SEM.

## 3. Results and Discussion

### 3.1. Microstructure Characterization

**Figure 2:**
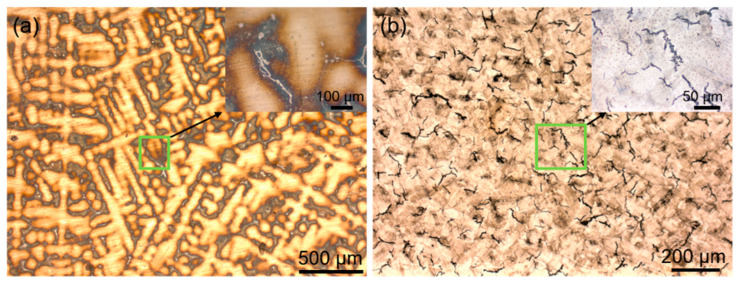
Optical microstructure images of K213 alloy. (**a**) As-cast, (**b**) SLM.

**Figure 3:**
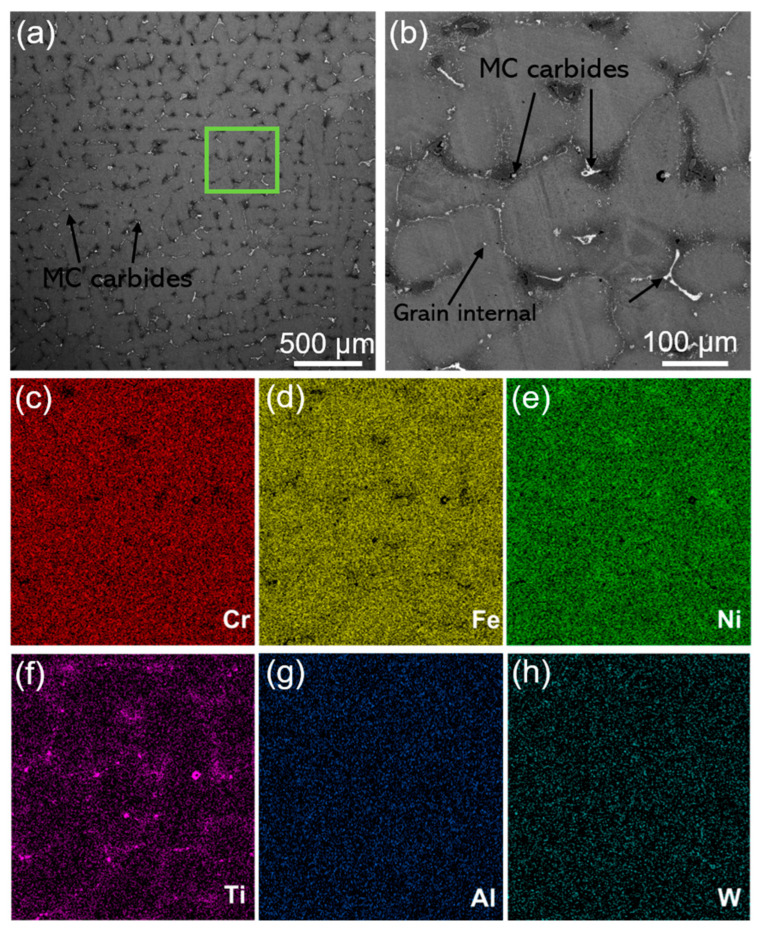
(**a**) Microstructure of as-cast K213 alloy. (**b**) Local magnified area of the green box in (**a**). (**c**–**h**) Distribution maps of corresponding main components.

Figure 2 shows the optical microstructure of the as-cast and SLM-formed K213 alloy. It can be seen from Figure 2a that the as-cast microstructure is dendritic; the enlarged area in the upper right corner shows white precipitates at the grain boundaries. The microstructure shown in Figure 2b (the SLM sample) is very different from that of the as-cast sample, and it is clear that there are many cracks. Figure 3 shows the SEM image of the microstructure of the as-cast K213 alloy and the corresponding distribution characteristics of Cr, Fe, Ni, Ti, Al, and W. It can be seen that the as-cast alloy has a typical dendrite microstructure, and there are a large number of white precipitates at the grain boundaries (Figure 3b). The essential reason for this phenomenon is the formation of primary MC carbides during melting. It can be seen from the composition distribution map that the content of Ti is higher at the grain boundaries, which also confirms the formation of MC carbide. A small amount of carbide is also formed inside the grains. The results show that these elements have a high tendency to segregate to the grain boundary, which is beneficial to the formation of liquid phase on the grain boundary in the melting process. However, the presence of carbides generally reduces the strength of the alloy, thereby initiating premature failure [23,24].

**Figure 4:**
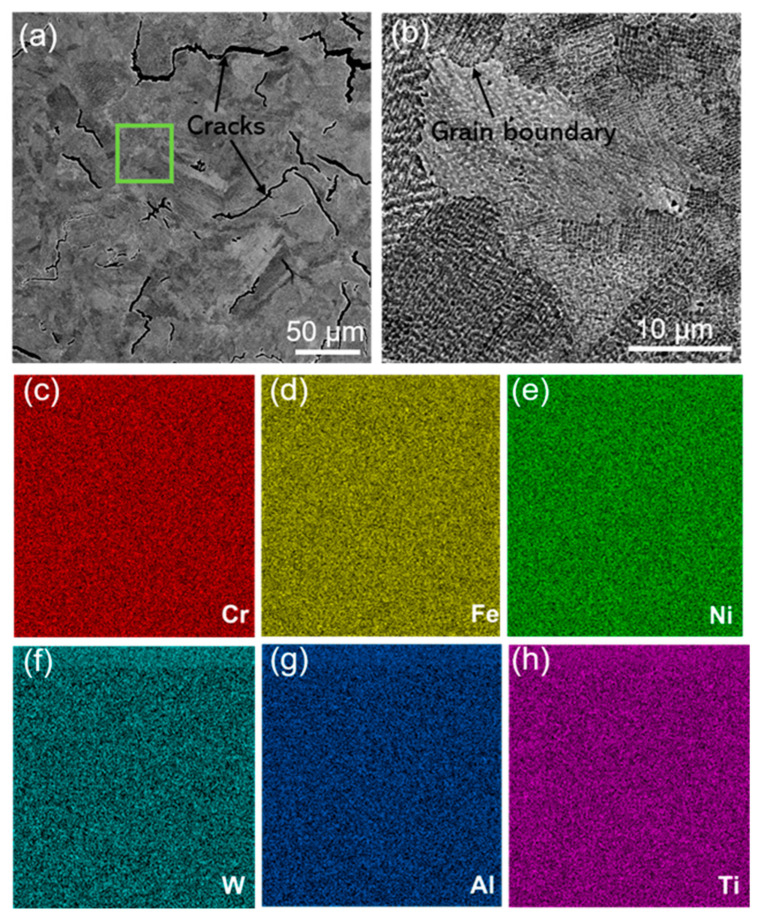
(**a**) Microstructure of SLM K213 alloy. (**b**) Local magnified area of the green box in (**a**). (**c**–**h**) Distribution maps of corresponding main components.

Figure 4 shows an SEM image of the microstructure of the SLM K213 alloy sample perpendicular to the deposition direction and corresponding composition distribution maps. It can be observed in Figure 4a that there are many cracks. The crack length is less than 200 μm, and the crack width is less than 10 μm. It is well-known that the cracking of alloys is determined by mechanical driving force against and the inherent resistance to cracking. The mechanical driving force generally refers to the stress or strain produced by rapid thermal cycling. Cracking resistance refers to the degree of intergranular ductility. The formation of cracks in SLM alloys is due to the presence of a temperature gradient in the direction perpendicular to the molten pool during deposition. The temperature gradient transfers heat to the solidified structure, resulting in higher residual stresses due to the faster cooling rate. The residual stress is distributed in the eutectic region, and it easily forms microcrack sources that propagate [25]. In addition, the formation of cracks in the SLM process may be related to the chemical composition, particle size, and fluidity of the alloy powder, as well as to the absorption rate and forming rate of the powder reacting to the laser in the SLM process. Different from the as-cast K213 alloy, the main components of the SLM-formed K213 alloy are uniformly distributed, without segregation at the grain boundaries.

### 3.2. Mechanical Properties

**Figure 5:**
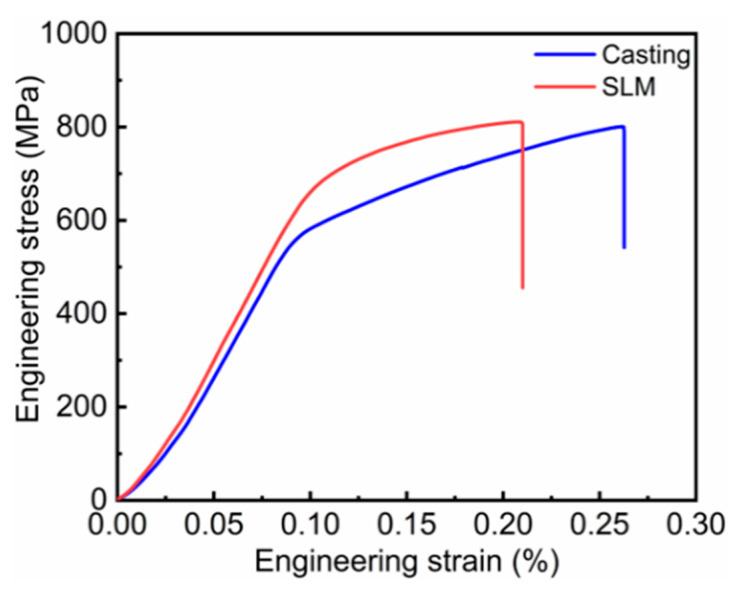
Engineering stress–strain tensile curve for the as-cast and SLM samples.

Figure 5 shows the tensile engineering stress–strain curves of the as-cast and SLM-formed specimens at room temperature. It can be seen from the figure that the tensile yield strength, ultimate yield strength, and elongation of the SLM sample are 680 MPa, 810 MPa, and ~21%, respectively. The tensile yield strength and ultimate yield strength of the SLM samples are higher than those of the as-cast samples. Notably, the SLM tensile yield strength is 100 MPa higher than that of the as-cast samples. However, the elongation of the as-cast sample is 26% greater than that of the SLM sample. In general, the as-cast specimens have a relatively good combination of strength and elongation.

**Figure 6:**
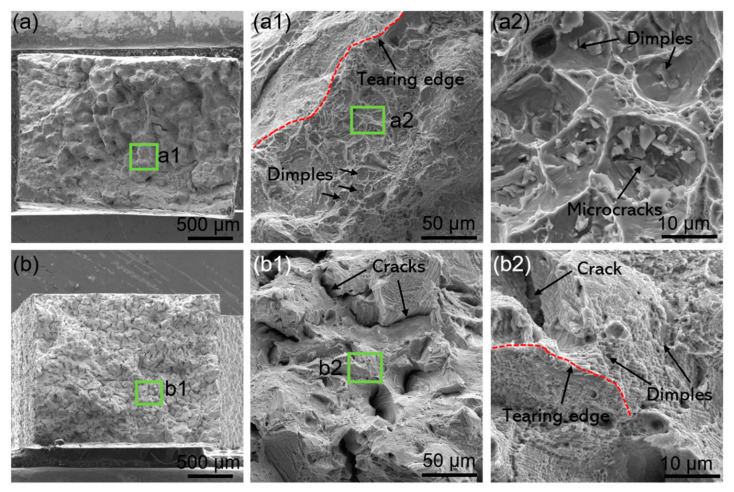
SEM images of tensile fracture surface for K213 alloy. (**a**) As-casting, (**a1**) Local magnified area of the green box in (**a**), (**a2**) Local magnified area of the green box in (**a1**). (**b**) SLM, (**b1**) Local magnified area of the green box in (**b**), (**b2**) Local magnified area of the green box in (**b1**).

The SEM morphologies of the room-temperature tensile fracture surfaces of the differently formed K213 alloys are shown in Figure 6. A significant difference can be observed between the two samples. The fracture surface of the as-cast sample shows a large number of bulges under a low-magnification microscope (Figure 6a). An enlargement of the typical position (green box in the figure) is shown in Figure 6a1. It can be observed that there are a large number of dimples in the middle of the bulge, and on the edge of the bulge is an obvious tear ridge. There are microcracks at the bottom of some dimples of the as-cast samples. The tensile fracture morphology of the SLM specimen (Figure 6b) is different from that of the as-cast specimen. Under low magnification, the surface of the fracture is less bulged. Similarly, it can be seen by magnifying the typical position (Figure 6b1,b2) that there are numerous cracks on the fracture surface. This is due to cracks formed in the matrix during the SLM-forming process. Stress concentration easily occurs in the crack area, which leads to the early fracture. The dimples and tear edges of the SLM sample are smaller than those of the as-cast alloy sample.

During the tensile deformation of the as-cast K213 alloy, there is a deformation incompatibility between the MC precipitates and the alloy matrix. Therefore, microvoids are formed between the second-phase particles and the matrix. With the further increase in deformation, the microvoids grow up, aggregate, and penetrate until the alloy fractures, thus forming dimples at the fracture surface. The results show that the MC caused by element segregation in the as-cast alloy is the main reason for the decrease in strength. On the other hand, the SLM K213 alloy without precipitates exhibits higher strength during tension; the inherent cracks are the main reason for limiting its further increase in strength and reduction of plasticity.

### 3.3. High-Temperature Oxidation Resistance

**Figure 7:**
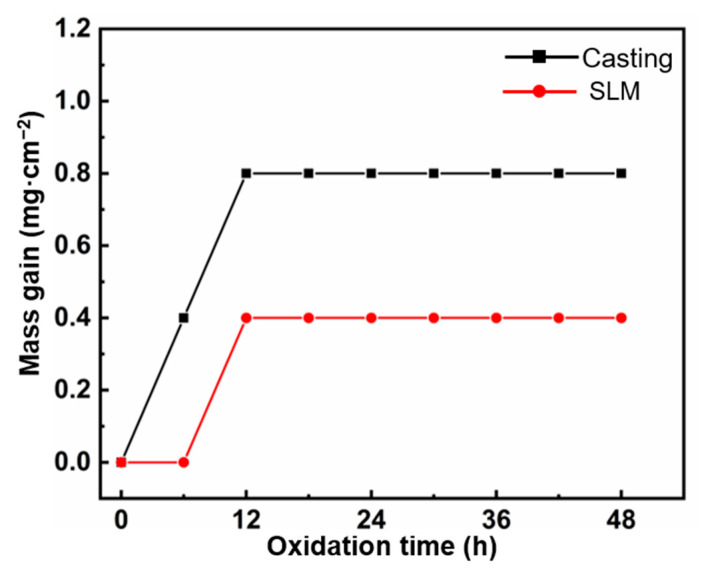
Recorded mass gain as a function of time during isothermal oxidation.

Figure 7 shows a curve of oxidation weight gain per unit area for as-cast and SLM specimens under high-temperature oxidation conditions at 800 °C. It can be seen that the weight gain curves of the two samples are significantly different. The weight of the as-cast sample rapidly increases in the range of period 0–12 h, reaching a value of 0.8 mg·cm^−2^ and then entering a stable stage. This may be due to the formation of an oxide film on the surface, which hinders the further oxidation of the substrate. However, there was no weight gain of the SLM sample within 0–6 h, indicating that oxidation did not occur within that time span. In the range of 6–12 h, the oxidation rate of the SLM sample is similar to that of the as-cast sample. Generally speaking, SLM samples have good high-temperature oxidation resistance.

**Figure 8:**
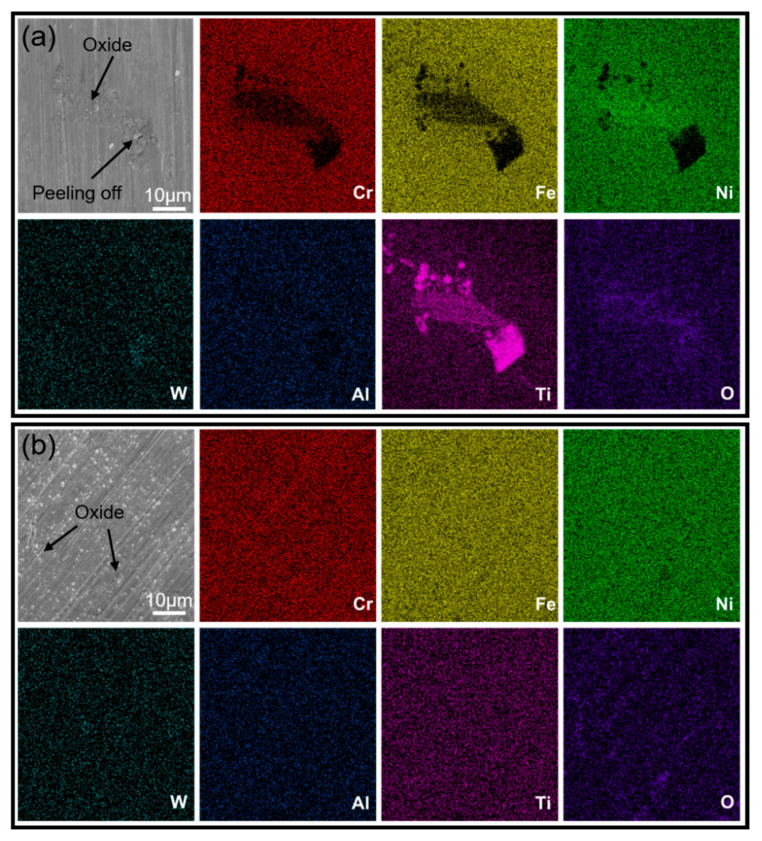
SEM images and mapping after oxidation at high temperature for 30 h. (**a**) As-cast, (**b**) SLM.

Figure 8 shows the SEM images of the as-cast and SLM samples after oxidation for 30 h and the corresponding composition distribution map. After oxidation at 800 °C for 30 h, the surface of the as-cast K213 alloy has been oxidized, and local severe oxidation has occurred. In the heavily oxidized area, the surface oxide layer is peeled off, as shown in Figure 8a. It can be seen from the composition distribution map that the surface of the as-cast alloy is basically fully covered by the O element. In the heavily oxidized region, Ti has high brightness, indicating that Ti has reacted with O. Combined with the above analysis of MC carbides, it can be concluded that the carbides in as-cast K213 will be more substantially oxidized than the matrix in the process of high-temperature oxidation, which will lead to the damage of the material surface. However, after oxidation of SLM samples for 30 h, the surface is uniformly oxidized and the surface material is intact, as shown in Figure 8b. The SLM sample has better high-temperature oxidation resistance than the as-cast sample within 30 h at 800 °C.

**Figure 9:**
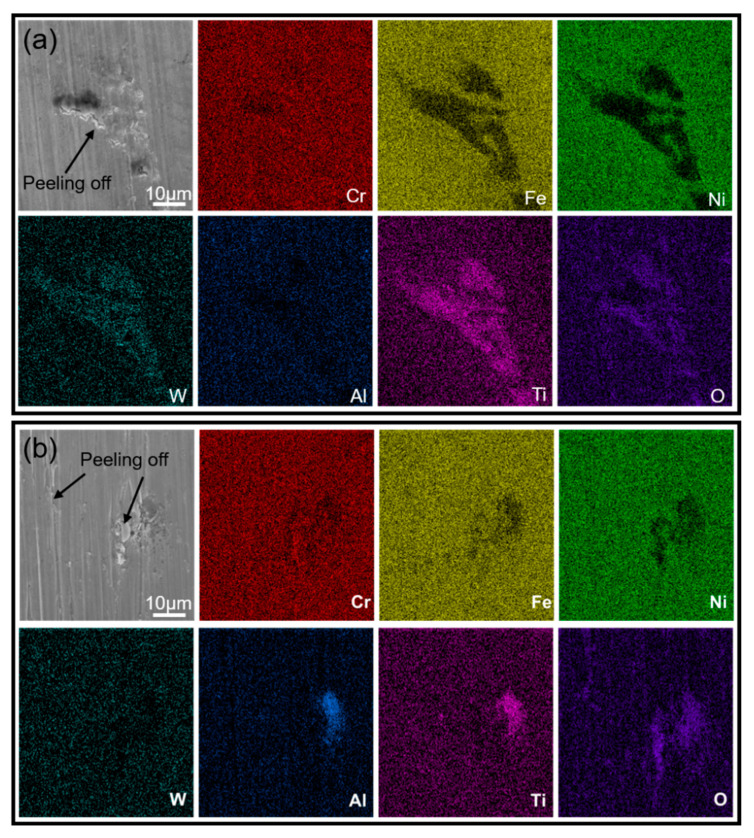
SEM images and mapping after oxidation at high temperature for 48 h. (**a**) As-cast, (**b**) SLM.

Figure 9 shows the SEM morphology and composition distribution of the two samples after oxidation at 800 °C for 48 h. The as-cast samples after oxidation for 48 h are similar to those after oxidation for 30 h. The spalling of the oxide scale also occurs on the surface of the as-cast K213 alloy. Severe oxidation also occurs in the region where MC carbides appear, as shown in Figure 9a. After oxidation at high temperature for 48 h, the surface material of the SLM sample is spalled. It can be seen from the composition distribution map that, unlike the as-cast sample, there are not only Ti and W but also Al in the significant oxidation area of the SLM sample.

### 3.4. Potentiodynamic Polarization Behavior

**Figure 10:**
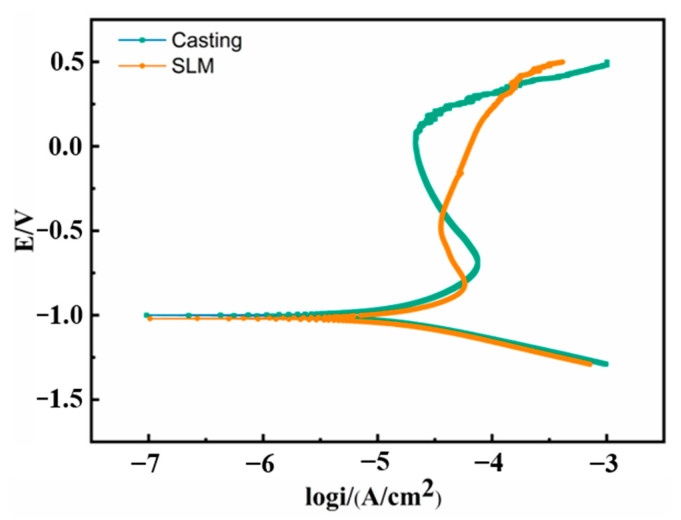
Polarization curves of as-cast and SLM samples.

Figure 10 shows the Tafel curves for the conventionally casted and SLM-formed specimens. The corrosion potential and corrosion current density corresponding to this experiment are shown in Table 2. The corrosion potential of the SLM specimen (−1.020 V) is lower than that of the as-cast alloy specimen (−1.000 V), which seems to indicate that the SLM specimen has a greater tendency to corrode, but this does not imply a true corrosion rate. The corrosion current density (Icorr) of the alloy was obtained by tangentially cutting the anodic and cathodic polarizations. The order of the Icorr of alloys is SLM (15.34 μA/cm^2^) > as-cast (11.72 μA/cm^2^). In general, the electrochemical corrosion resistance of the as-cast sample is slightly better than that of the SLM sample. This is due to the inherent cracks in the SLM specimen in the corrosive solution to induce the diffusion of corrosion into the matrix.

## 4. Conclusions

This study investigated the effect of microstructure on the mechanical and corrosion properties of conventionally cast and SLM-formed specimens. Several conclusions were reached.

(1) During the melting process of the K213 alloy, the segregation of Ti occurs in the grain interior and grain boundaries, and MC carbides are formed. However, the composition of the SLM K213 alloy is uniform, although cracks are formed in the matrix.
(2) The as-cast K213 alloy has a relatively good combined effect of strength and plasticity. The main factors limiting the mechanical properties of the as-cast and SLM K213 alloys are carbides and cracks, respectively. The reason is that there is deformation incompatibility between the carbides and matrix in the as-cast sample, and the cavity defect is easy to occur during the tensile process. Stress concentration is easy to occur in the crack area of the SLM specimen, which leads to early fracture.
(3) The SLM sample has relatively well high-temperature oxidation resistance. The MC precipitates in the as-cast K213 alloy accelerate the high-temperature oxidation. In addition, due to the different solidification segregations in the alloy, different oxides are formed during the oxidation process for the two samples. Cracks in the SLM alloy weaken the electrochemical corrosion resistance.

## Figures and Tables

**Table 1:** Composition of K213 alloy powder (wt.%).

Ni	Cr	Ti	W	Al	Mn	S	Si	C	Fe
37.25	15.13	3.88	5.365	1.585	0.078	0.007	0.143	0.061	Bal.

**Table 2:** Polarization test results for casting and SLM samples.

Samples	E_corr_ (V)	I_corr_ (μA/cm^2^)
Casting	−1.000	11.72
SLM	−1.020	15.34

## Data Availability

Data are contained within the article.
